# Twigs-derived activated carbons via H_3_PO_4_/ZnCl_2_ composite activation for methylene blue and congo red dyes removal

**DOI:** 10.1038/s41598-020-71034-6

**Published:** 2020-08-20

**Authors:** Muhammad Hadzirun Muhamad Zubir, Muhammad Abbas Ahmad Zaini

**Affiliations:** 1 Centre of Lipids Engineering and Applied Research (CLEAR), Ibnu-Sina Institute for Scientific and Industrial Research, Universiti Teknologi Malaysia, 81310 UTM Johor Bharu, Johor Malaysia; 2 School of Chemical and Energy Engineering, Faculty of Engineering, Universiti Teknologi Malaysia, 81310 UTM Johor Bahru, Johor Malaysia

**Keywords:** Pollution remediation, Chemical engineering

## Abstract

This work is aimed at evaluating the conversion of *Pterocarpus indicus* twigs into activated carbon via composite chemical activation for methylene blue and congo red dyes adsorption. The activated carbons were prepared by single-step chemical activation using zinc chloride and/or phosphoric acid at different mass impregnation ratios at 600 °C for 90 min. The activated carbons were characterized for textural properties and surface chemistry. The batch adsorption was investigated at different concentrations (5–1,000 mg/L), contact times (2–540 min) and temperatures (30–60 °C). Phosphoric acid-activated twigs carbon showed a high surface area of 1,445 m^2^/g with maximum methylene blue adsorption capacity of 438 mg/g. On the other hand, a composite-activated carbon yields a 217 mg/g of congo red removal. The adsorption data for both dyes fitted well with Langmuir and pseudo-second-order kinetics models, indicating the predominance of chemical adsorption through monolayer coverage of dye molecules on the homogenous surface of activated carbon. The thermodynamics properties of dye adsorption onto twigs-derived activated carbons indicated that the process is endothermic, spontaneous and favourable at high temperature. Overall, activated carbons derived from *Pterocarpus indicus* twigs could be effectively used for dye wastewater treatment.

## Introduction

In many developing countries worldwide, textile industry is among the vast economy-driven activities with 2.8% rise in demand every year^1^. However, it becomes a main contributor to water pollution issue that brings about multiple negative impacts to aquatic environment, ecological balance, and human health^2^. The fabric sector consumes nearly several hundred thousand gallons of water every day, hence generating a large quantity of wastewater^3^. Typical textile wastewater consists of nutrients such as phosphate, nitrates, micronutrients, and sources of carbon for algae cultivation^4^. Yet, it also primarily includes colorants and auxiliary chemicals which are toxic and harmful, hence creating negative implications to the water streams^5^.

Dyestuff components are toxic, carcinogenic, and mutagenic. Generally, dye is persistent for a long duration because the molecular structure is complex and can withstand degradation by sunlight and microorganism in water. Additionally, dye in water can impede the penetration of sunlight, thereby reducing the rate of photosynthesis and consequently results in oxygen deficiency for respiration^6^. Dyes can be categorized into anionic, cationic, and non-ionic. For example, methylene blue (basic blue 9) is among the prevalent cationic dyes used in the fabric sector and can cause damaging effects, primarily to the aquatic ecosystem and human health.

Wastewater treatment strategies have been introduced to mitigate water pollution. Among others, adsorption outweighs other removal techniques because the process is simple, economical, and feasible for dye wastewater decontamination even at low concentration depending on the physicochemical properties of adsorbent^6^. Activated carbon is a commonly used adsorbent in adsorption. It can be produced from cellulosic and carbonaceous materials through physical activation using air, CO_2_, and steam, or chemical activation using dehydrating agents such as ZnCl_2_, KOH and H_3_PO_4_. Chemical activation brings certain advantages over physical activation, because of lower activation temperature (450–700 °C), high activated carbon yield and high total surface area of activated carbon produced for effective water pollutants removal.

To date, the characteristics and adsorptive properties of activated carbons derived from abundantly available natural cellulose, namely *Pterocarpus indicus* twigs via composite chemical activation are not well documented, therefore worth to be investigated to broaden the body of existing knowledge. The exploitation of combined activating agents, as in this work, H_3_PO_4_ and ZnCl_2_ may contribute significant findings towards dyes adsorption from the viewpoints of surface chemistry and structure of activated carbons produced. Two commonly used dyes in textile industry, namely congo red (anionic dye) and methylene blue (cationic dye) were employed to establish the adsorption profiles of isotherm, kinetics, and thermodynamics to shed insight into industrial applications. The adsorption selectivity and governing mechanisms were also discussed.

## Materials and methods

*Pterocarpus indicus* twigs was collected in UTM Johor Bahru campus. It was cut to a size of 1.5–2.0 cm in length and dried in an oven (UFB-400, Memmert, Germany) at 110 °C for 12 h to remove moisture. Zinc chloride and methylene blue dye were purchased from HmbG Chemicals (Hamburg, Germany), while H_3_PO_4_ was obtained from R&M Chemicals (Essex, UK). Hydrochloric acid sodium hydroxide and congo red dye were purchased from Merck (Darmstadt, Germany). All chemicals are of analytical grade reagents.

### Preparation of twigs-based activated carbon (TAC)

The preparation of activated carbons (TACs) involved chemical impregnation, carbonization, and activation. Twigs was mixed with ZnCl_2_ and/or H_3_PO_4_ at different mass ratios as summarized in Table 1. The solid-solution mixture was stirred continuously at 70 °C for 90 min. Next, it was dried in an oven at 110 °C for 24 h for impregnation. The impregnated sample was introduced in a muffle furnace under anoxic environment for single-step activation at 600 °C for 90 min. After that, the resultant carbon was soaked in dilute HCl solution overnight for partial demineralization. Then, it was washed using distilled water until the pH of activated carbon remains unchanged. Finally, the activated carbon was oven-dried prior to use. The yield of activated carbon was determined from the product mass divided by the mass of twigs used in activation.

**Table 1:** Impregnated materials and activated carbons derived from *Pterocarpus indicus* twigs.

Mass ratio			Sample coding	
Twigs raw material (TRM)	ZnCl_2_	H_3_PO_4_	Impregnated material	Activated carbon
1	0	0	imTRM1	TAC1
1	1	0	imTRM2	TAC2
1	0	1	imTRM3	TAC3
1	0.5	0.5	imTRM4	TAC4
1	0.25	0.75	imTRM5	TAC5
1	0.75	0.25	imTRM6	TAC6

### Characterization of TAC

Thermogravimetric analysis (TGA) was performed using a TGA-Q500 equipment (TA Instruments, USA). The temperature was ramped from room temperature to 900 °C, at a heating rate of 10 °C/min under N_2_ flow. The elemental composition was determined using a Vario Micro Cube analyzer (Elementar, Germany). The combustion was performed at 1,150 °C by injecting O_2_ gas into the sample chamber. The surface composition and morphology were obtained by a SEM–EDX integrated machine (TM3000, Hitachi, Japan). The textural properties of activated carbon were determined at liquid N_2_ temperature of 77 K using a surface area analyzer (ASAP2020, Micromeritics, USA).

The peaks of functional groups of activated carbon were recorded by attenuated total reflectance FTIR Spectrometer (Spectrum One, PerkinElmer, USA). Boehm titration was carried out to characterize the quantitative acidic and basic groups in activated carbon. The sample of 0.3 g was added into 15 mL of different solutions of 0.1 M NaOH, 0.1 M HCl, 0.1 M NaHCO_3_ and 0.05 M Na_2_CO_3_. The mixtures were allowed to stay for 48 h. Then, 5 mL of supernatant was taken out for back-titration. The excess acid was titrated with 0.05 M HCl solution, while the excess base was titrated with 0.1 M NaOH. Phenolphthalein and methyl red were used as pH indicators. The pH of the point of zero charge (pH_pzc_) is a feature to determine the surface charge of activated carbon in the solution. A 0.1 g sample was added to flasks containing 50 mL of 0.1 M NaCl solution at varying pH between 2.5 and 10.5. The pH was initially adjusted using drops of 0.1 M HCl and 0.1 M NaOH. The mixtures were allowed to equilibrate for 24 h, and the final pH was measured using a pH meter (HI 8,424, Hanna Instruments, UK).

### Dyes adsorption studies

Fifty mg of activated carbon was added into a series of flasks bearing 50 mL of dye solution of varying concentrations from 5 to 1,000 mg/L. The solid-solution system was allowed to equilibrate for 72 h at room temperature. The change in solution pH was monitored and recorded. The residual concentration was measured using a UV–visible spectrophotometer (DU8200, Drawell Scientific, China) at wavelengths of 600 nm and 490 nm for methylene blue and congo red, respectively. The adsorption capacity at equilibrium, *Q*_*e*_ (mg/g) was calculated as,

1 $${Q}_{e}=\frac{{(C}_{o}-{C}_{e})V}{m}$$

where *C*_*o*_ and *C*_*e*_ (mg/L) are initial concentration and equilibrium concentration, respectively, *V* (L) is the solution volume and *m* (g) is the mass of activated carbon.

Similar settings were repeated for adsorption kinetics. Fifty mg of activated carbon was added to flasks containing 50 mL of dye solution with concentrations of 20 and 100 mg/L. The system was allowed to stay at ambient temperature and the concentration was measured at different time intervals between 2 and 540 min. The adsorption capacity at time *t*, *Q*_*t*_ (mg/g) was calculated as,

2 $${Q}_{t}=\frac{{(C}_{o}-{C}_{t})V}{m}$$

where *C*_*t*_ (mg/L) is the concentration at time, *t*.

The thermodynamic parameters were determined from the equilibrium adsorption at different temperatures from 30 to 60 °C. The concentrations of 20 and 100 mg/L were selected to represent the adsorption conditions. The pH was left unadjusted for the entire adsorption process. All experiments and measurements were reproduced in duplicate and the average values were reported.

### Models fitting

The adsorption data were analyzed using adsorption models to describe the transport behaviour and removal mechanisms. The isotherm and kinetic models and thermodynamic parameters are summarized in Table S1. The non-linear equations were solved using Microsoft Excel solver.

## Results and discussion

### Characteristics of activated carbon

#### Proximate analysis

**Figure 1:**
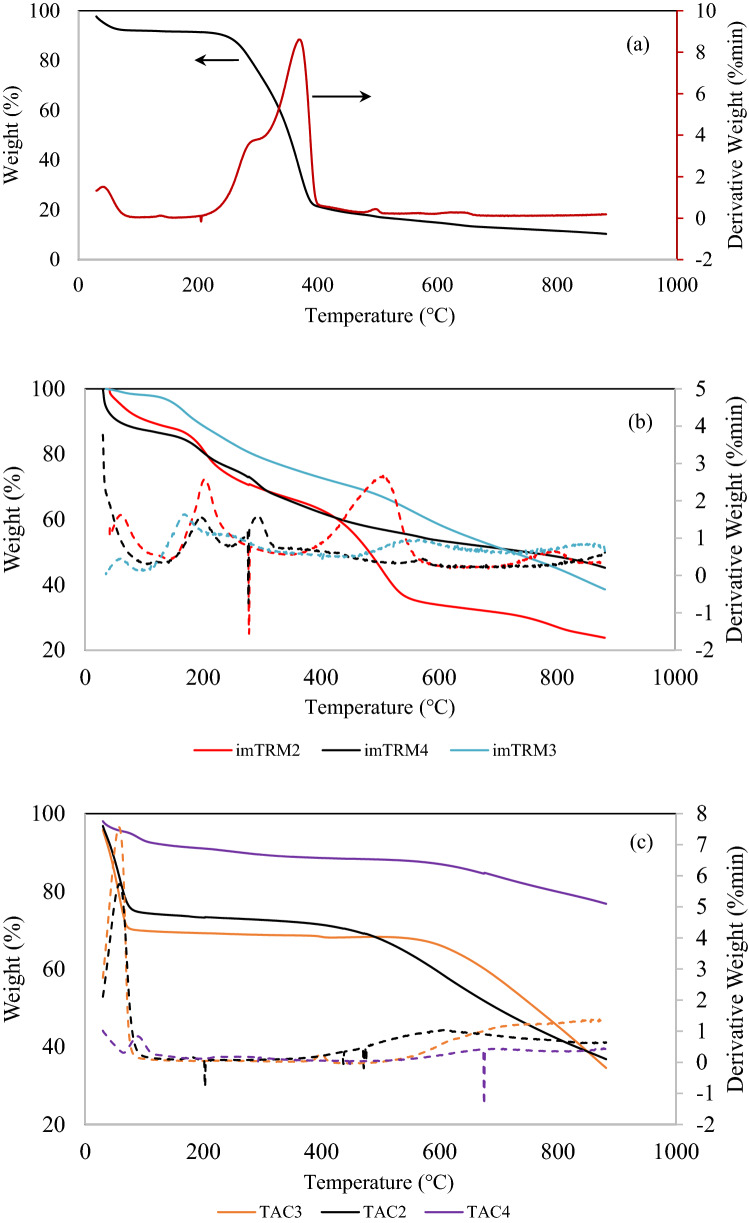
Thermal degradation profiles of (**a**) TRM, (**b**) impregnated materials and (**c**) activated carbons.

Figure 1a shows the thermal degradation profile of twigs (TRM). There is a decrease in weight from 100 to 92% at temperature 105 °C due to the release of moisture and dehydration of water. The existence of thermally stable constituents can create a little weight loss in material at 300 °C^7^. The heat resistance of twigs material is due to semi-crystalline arrays of hemicellulose, which forms strong connected chains. Also, the polysaccharide chains in lignin can create a complex structure of phenolic and aromatic groups in the cell wall structure. These combinations render a durable and heat-resistant material^8^. At 375 °C, the material undergone major decomposition, wherein the weight drastically decreased from 90 to 20% due to the liberation of volatiles from semi-cellulose and lignin. Semi-cellulose is relatively easy to decompose because of linear polymer structure and short side chains^9^. The gradual weight loss at a temperature range of 400–900 °C can be attributed to the decomposition of heavy organic materials. The carbon-rich matrix is intensely gasified into CO and CO_2_, leaving behind the ash content of 10%. From the TGA analysis, the fixed carbon and volatile content were recorded as 10% and 72%, respectively. Also, a suitable temperature range for activation is around 400–600 °C.

Figure 1b shows the thermal degradation profiles of impregnated samples (imTRMs). Sample imTRM2 shows a weight loss of 10% at 100 °C due to the elimination of moisture content. The estimated value of moisture content is slightly higher than that of TRM because of the presence of ZnCl_2_ that is hygroscopic, i.e., able to adsorb more moisture from the surrounding. The maximum weight loss of imTRM2 at 200 °C indicates the decomposition of hemicellulose and cellulose. The degradation at 500 °C could be referred to the devolatilization of lignin and residual char. A rapid degradation of imTRM2 produces a high residue of 23%, which consists of fixed carbon and zinc element. This is because, ZnCl_2_ could inhibit the formation of tar, hence preserving the yield^10^. The evaporation of ZnCl_2_ could occur at a temperature range of 400–600 °C^11^. Aromatic condensation may also take place, resulting in the evolution of gaseous products due to the presence of hydro-aromatic structure in the raw material. Sample imTRM3 shows a weight loss nearly 5% at 95 °C, which is slightly lower than that of TRM due to H_3_PO_4_ activation. A weight loss of 5–20% between 150 and 250 °C is due to the elimination of volatile matters. The gradual decomposition of light organic constituents indicates that H_3_PO_4_ restricts the volatilization of carbon, leading to a high fixed carbon of 26%^12^. A 40% of ash content is likely from the phosphorus residues of activating agent. The moisture content of imTRM4 is quite similar with that of TRM. The combination of chemical reaction of ZnCl_2_ and H_3_PO_4_ in imTRM4 produces zinc phosphate cement, Zn_3_(PO_4_)_2_ as shown in Eq. (3). At 100–230 °C, some hydrated zinc phosphate salts (Zn_3_(PO_4_)_2_·4H_2_O) such as hopeite formed after the evolution of cement during the water evaporation^13^.

3 $$3{\mathrm{ZnCl}}_{2} (\mathrm{aq}) + 2{\mathrm{H}}_{3}{\mathrm{PO}}_{4} (\mathrm{aq})\rightarrow {\mathrm{Zn}}_{3}{\left({\mathrm{PO}}_{4}\right)}_{2} (\mathrm{s}) + 6\mathrm{HCl }(\mathrm{aq})$$

Figure 1c shows the TGA profiles of activated carbons. The thermal degradation trends of TAC2 and TAC3 are almost similar. The moisture content due to the elimination of water for TAC2 and TAC3 are 23% and 28%, respectively. TAC3 shows excellent thermal stability that can withstand up to 600 °C, as compared to TAC2 which is only up to 480 °C. H_3_PO_4_ in TAC3 promotes the reducing of weight loss of carbon due to the formation of phosphate bonds which cross-linked with each other to form the polyphosphate bridges^14^. Consequently, it promotes the development of porous structure at wider activation temperature. The ash content of TAC2 and TAC3 are comparable at 38% and 35%, respectively. The TAC2, TAC3 and TAC4 gave the volatile matter of 4, 2 and 6%, respectively, reflecting the high stability of activated carbons at low temperature. The formation of hopeite in TAC4 reduces the melting point so that the mass of ZnCl_2_ remains in char. Thus, the ash content of TAC4 is slightly higher than that of TAC2 and TAC3, which is 76%. The values of volatile matter, fixed carbon, moisture content and ash content from the TGA data are given in Table S2.

The yield of TAC1 (char) is 21.3%, which is smaller than other TAC samples because there is no chemical impregnation involved, leading to high carbon and volatiles released. The yields of TAC2 and TAC3 are 41.3% and 50%, respectively. Zinc chloride acts as a dehydrating agent to enhance the removal of volatiles such as H_2_ and O_2_ from the carbon matrix. It exhibits a high boiling point of 732 °C, and highly soluble in water which accelerates the removal of water and volatile molecules. A high amount of ZnCl_2_ can accelerate the evaporation of volatiles through the breakdown of aromatic and aliphatic chains. Nevertheless, ZnCl_2_ can preserve the carbon content via the formation of long aromatic chain structure composed primarily of graphitic structure of carbon atoms, resulting in a high yield of TAC2 as compared to that of TAC1. It comprises of networks of pores with channels constructed within a rigid skeleton of disordered layers of carbon atoms, connected by chemical bonds and stacked unevenly. ZnCl_2_ also acts in pore drilling that enters the carbon matrix to form large pore volume and high surface area of activated carbon, while at the same time releasing some volatiles^10^.

The high percentage yield of TAC3 compared to TAC2 is due to the residual phosphorus element present after the activation process because. The phosphate residue from H_3_PO_4_ activation is water insoluble. The chemical reaction between H_3_PO_4_ and lignocellulosic materials like hemicellulose and lignin during activation creates weak hydrolyze glycosidic linkages, thus accelerating the decomposition process. However, cellulose is not easily broken down because it acts as acid hydrolysis resistant material, leading to the high yield of TAC3^15^.

#### Elemental composition

Elemental composition of a material was determined through dynamic flash combustion technique. Table 2 shows the elemental composition of raw material and activated carbons. The presence of oxygen content in TACs can be attributed to the oxidation process which occurred during the activation and after the materials are exposed at room temperature. According to^16^, oxygen molecules are localized at the carbon surface and form electrostatic interactions with the positively charged functional groups. A smaller fraction of oxygen in TAC2 (35%) and TAC3 (18.8%) is due to rapid removal of volatiles during the activation at high temperature, thus leaving behind reactive oxygen element.

**Table 2:** Elemental composition of TRM and its derived activated carbons.

Element (%)	TRM	TAC1	TAC2	TAC3
Carbon	46.3	32.9	62.3	78.6
Hydrogen	6.87	1.60	2.17	1.84
Nitrogen	0.265	0.197	0.492	0.615
Oxygen	46.5	65.2	35.0	18.8
Sulphur	0.041	0.068	0.077	0.095

The elements of carbon, oxygen, and hydrogen make-up the building matrix of TRM which contains lignin, cellulose, and hemicellulose. Some of these constituents are easily decomposed at high temperature. As a result, the carbon content in char TAC1 decreased to 32.9%. Through suitable activation strategies, TRM could be a potential precursor for activated carbon production. Table S3 summarizes the comparison of elemental composition of TRM with other lignocellulosic raw materials of activated carbon in literature.

**Figure 2:**
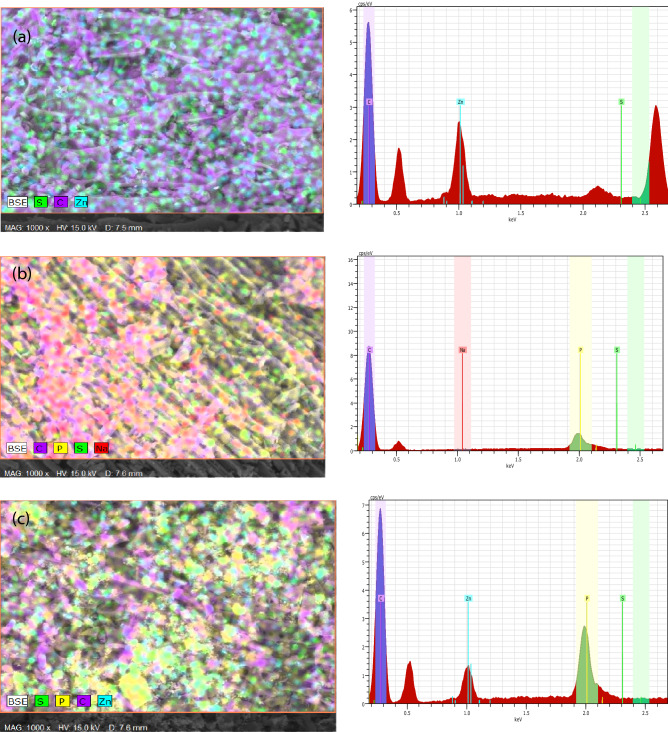
EDX mappings and spectra of (**a**) TAC2, (**b**) TAC3 and (**c**) TAC5.

Figure 2 shows the EDX mappings and spectra of activated carbons. The distribution of carbon is dominant, with scattered elements of zinc, phosphorus and sulphur. Table 3 shows the elemental composition on the external surface of activated carbons. The surface of TAC2 consists of 81.1% carbon, 4% zinc and 0.47% sulphur. The presence of zinc and sulphur could be originated from ZnCl_2_ used in activation and sulphur content in organic precursor, respectively. The oxidation process also prompts the formation of zinc oxide, ZnO deposited on TAC2. The formation of ZnO is based on the reaction between ZnCl_2_ and oxygen released from the organic material as shown in Eq. (4). The ZnO produced acts as oxygen-soluble material, but insoluble in water.

**Table 3:** EDX surface analysis of activated carbons.

Activated carbon	Mass of element (wt%)				
	Carbon	Zinc	Phosphorus	Sulphur	Sodium
TAC2	81.1	4.00	–	0.47	–
TAC3	95.3	–	4.41	0.13	0.14
TAC5	73.6	11.6	14.7	0.10	–

4 $${\mathrm{ZnCl}}_{2} (\mathrm{s}) + 0.5{\mathrm{O}}_{2} (\mathrm{g}) \rightleftharpoons \mathrm{ZnO }(\mathrm{s}) + {\mathrm{Cl}}_{2} (\mathrm{g})$$

TAC3 displays a higher carbon content of 95.3%. A high carbon composition is imperative in the formation of smooth surface and well-developed porous structure of activated carbon. The presence of phosphorus is attributed to the use of H_3_PO_4_ in activation^17^. The sodium (0.14%) residue could be resulted from the impurity during the washing step. TAC5 exhibits a smaller carbon content than TAC2 and TAC3. The formation of zinc phosphate increases the fractions of zinc and phosphorus elements, thereby decreasing the carbon content.

#### Morphology and textural properties

**Figure 3:**
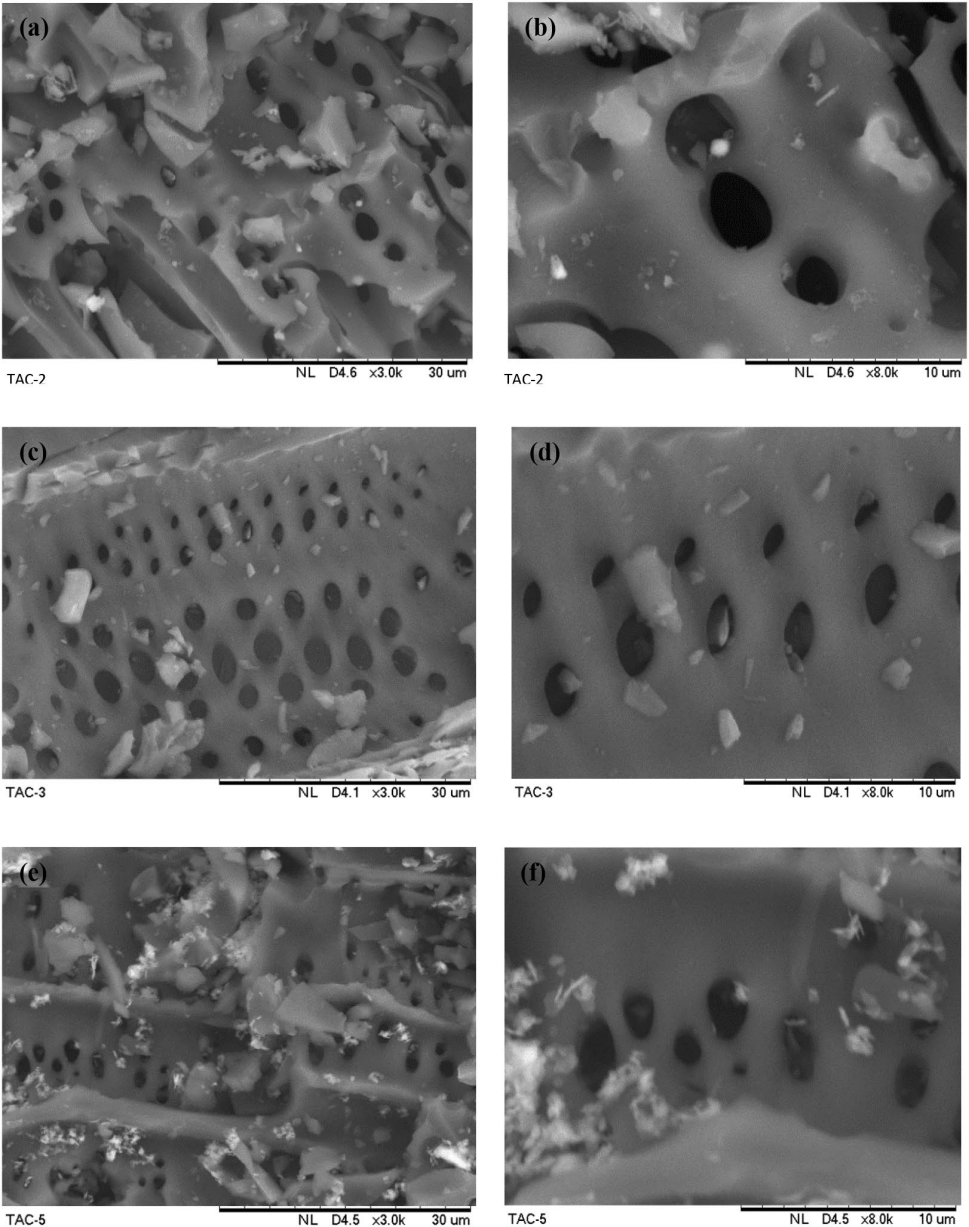
SEM images of TACs at different magnifications: (**a**, **b**) TAC2, (**c**, **d**) TAC3 and (**e**, **f**) TAC5.

Figure 3 shows the morphology of activated carbons at different magnifications. Figure 3a,b shows the SEM images of TAC2. TAC2 exhibits a surface with large pore volume. The development of pores within the matrix of TAC2 is associated to the evaporation of volatiles in the presence of dehydrating agent, ZnCl_2_. Nevertheless, insoluble oxides can remain on TAC2 surface due to oxidation at activation temperature that is below the boiling point of ZnCl_2_ of 732 °C, thus blocking some pathways^10^.

The smooth surface with more structured and ordered pores arrangement could be observed from the morphology of TAC3 as shown in Fig. 3c,d. Although insoluble phosphorus deposits may present, TAC3 demonstrates the smooth development of pores compared to TAC2 and TAC5. This is due to the evaporation of phosphoric acid during carbonization process, leaving behind the space formerly occupied by the activating agent.

Figure 3e,f shows the SEM images of TAC5. Based on the topography view, the insoluble Zn_3_(PO_4_)_2_ deposits scatter and clog the pore openings. The high melting point of zinc phosphate (900 °C) as opposed to the activation temperature at 600 °C could be the reason for its distribution over the carbon surface This situation can hamper the diffusion of molecules in adsorption due to inaccessible pores.

The yield, pH, pH_pzc_ and textural properties of activated carbons are summarized in Table 4. Figure S1 shows the N_2_ adsorption–desorption isotherm and pore size distribution of TACs. The decreasing order of BET surface area is TAC3 (1,445 m^2^/g) > TAC2 (907 m^2^/g) > TAC5 (498 m^2^/g) > TAC6 (394 m^2^/g) > TAC4 (344 m^2^/g) > TAC1 (190 m^2^/g). In the absence of activation, char (TAC1) displays a smaller surface area. A relatively smaller surface area of TAC4, TAC5 and TAC6 as opposed to TAC2 and TAC3 is due to the formation of ZnO, hopeite, and parahopeite minerals that partly clog the pores.

**Table 4:** Yield, pH, pH_pzc_ and textural properties of activated carbons.

	TAC1	TAC2	TAC3	TAC4	TAC5	TAC6
Yield	21.3	41.3	49.9	73.1	59.7	52.8
pH	7.30	6.90	4.90	5.80	5.30	7.20
pH_pzc_	7.90	6.25	3.30	5.30	3.85	6.40
BET surface area (m^2^/g)	190	907	1,445	344	498	394
Micropore surface area (m^2^/g)	139	266	565	293	242	300
External surface area (m^2^/g)	50.9	641	880	51.9	256	94.6
Langmuir surface area (m^2^/g)	254	1,240	1976	455	669	524
Total pore volume (cm^3^/g)	0.196	0.450	0.730	0.169	0.325	0.273
Micropore volume (cm^3^/g)	0.065	0.111	0.237	0.136	0.110	0.139
Mesopore volume (cm^3^/g)	0.132	0.339	0.493	0.033	0.215	0.134
Mesoporosity (%)	67.3	75.3	67.5	19.5	66.2	49.1
Average pore size (nm)	4.13	1.98	2.02	1.96	2.61	2.77

The Langmuir surface area and total pore volume signify the pore development of activated carbon. The total pore volume of TAC3 and TAC2 are 0.730 cm^3^/g and 0.450 cm^3^/g, respectively. A high total pore volume can be attributed to the formation of pores and pathways within the matrix of activated carbon. Furthermore, H_3_PO_4_ in TAC3 preparation has two important roles, (1) to increase the rate of thermal decomposition of raw material, and (2) to promote the formation of cross-linked bonding^18^. Table S4 shows the comparison between TAC3 and other activated carbons from the viewpoint of textural properties.

The adsorption behaviour of activated carbon depends not only on its inner surface area but also the pore size. TAC2, TAC3 and TAC4 are microporous with pore width of 1.98 nm, 2.0 nm 1.96 nm, respectively. Meanwhile, TAC1, TAC5 and TAC6 are mesoporous with pore width of 4.13 nm, 2.61 nm and 2.77 nm, respectively. It shows that H_3_PO_4_ instigates the development of micropores in TAC3 and TAC4 and mesopores in TAC5 and TAC6. The underdeveloped porous texture of TAC1 indicates that it is not suitable for adsorption because of the restricted access and limited active sites, even though it is highly mesoporous.

#### Surface chemistry and functional groups

**Figure 4:**
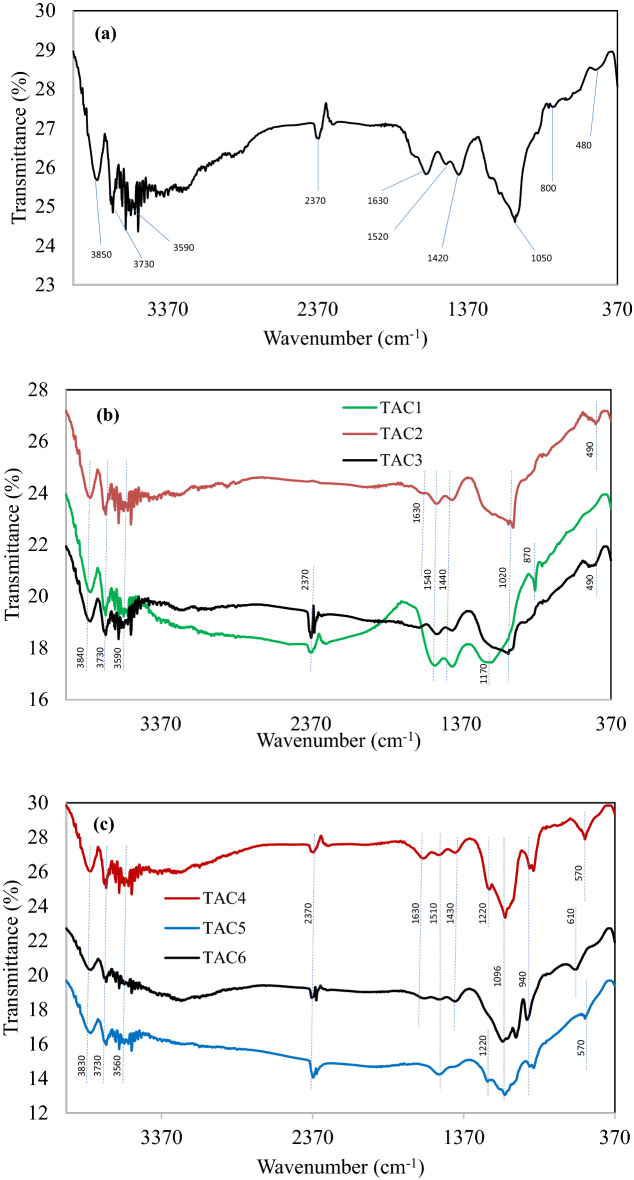
FTIR spectra of (**a**) TRM and (**b**) TAC1, TAC2 and TAC3, and (**c**) TAC4, TAC5 and TAC6.

The FTIR spectra of raw material and activated carbons are shown in Fig. 4. The peak at 3,850–3,700 cm^−1^ is due to physisorbed H_2_O molecules^19^. All samples display identical band of medium intensity around 3,500 cm^−1^ which is attributed to the vibration of hydroxyl groups (OH) such as alcohol and phenol due to hydrogen bonding from water absorption, and primary amines (NH) stretching^20^. The presence of hydroxyl groups is associated with the lignocellulosic structures in the parent material. The band of 2,370 cm^−1^ indicates the stretching of carboxylic groups (COOH). However, it disappeared in TAC2 spectrum because of strong dehydrating effect of ZnCl_2_ during activation.

The alkene (C=C) stretching vibration is the characteristic of peak at 1,640 cm^−1^ in TRM spectrum. The vibration probably occurred in olefinic and acyclic compounds, and conjugates. The profile is closely related to lignocellulosic-rich mustard card as reported in^21^. Yet, the peak is obviously diminished in all heated samples due to the conversion of aliphatic long chain to graphene (aromatic) sheets. The multiple bands between 1,500 and 1,400 cm^−1^ could be assigned to C=C stretching in aromatic groups or benzene rings and the bending vibration of methylene (alkane) groups.

The peaks at wavenumber below 650–450 cm^−1^ are generally attributed to C-X bonds where X could be C, O and N atoms for volatile matters such as sulphur, silicone, and halogenated compounds. The weak peaks around 950–800 cm^−1^ signify the CH vibration of alkenes and compounds derived from benzene that is associated with the out-of-plane deformation from the bending of CH and hydroxyl groups. The strong intensity and broad peak around 1,100–1,010 cm^−1^ in all spectra indicates strong absorption of CO groups of primary alcohols (R-OH), phenol groups and asymmetric vibration of Si–O–Si. Specifically, the peaks at 950–800 cm^−1^ are related to asymmetric vibration of Si–OH and symmetric stretching vibration of Si–O–Si.

Table 5 shows the concentrations of acidic and basic groups in activated carbons by Boehm titration. According to^22^, the basic nature is mainly due to oxygen-containing groups like carbonyl, ether, diketone and chromene, delocalized of π-electrons of carbon basal planes, and inorganic minerals. While, the acidic nature is due to the presence of carboxylic, lactonic and phenolic groups.

**Table 5:** Concentration of surface functional groups by Boehm titration.

Activated carbon	Carboxylic groups (mmol/g)	Lactonic groups (mmol/g)	Phenolic groups (mmol/g)	Total acidic sites (mmol/g)	Total basic sites (mmol/g)
TAC1	0.20	0.05	0.10	0.35	0.761
TAC2	0.30	0.00	0.35	0.65	0.217
TAC3	0.70	0.00	0.45	1.15	0.109
TAC4	0.25	0.15	0.40	0.80	0.000
TAC5	0.50	0.00	0.35	0.85	0.000
TAC6	0.35	0.00	0.25	0.60	0.326

TAC3 shows a total acidic concentration of 1.15 mmol/g. TAC4 and TAC5 exhibit the absence of basic sites, while TAC1 endows a 0.761 mmol/g of basic groups. The presence of acidic and basic groups in activated carbons are in accordance with the peaks in FTIR spectra. Basically, the density of acidic and basic sites is due to the nature of *Pterocarpus indicus* twigs and chemical activation strategies. The presence of surface functional groups is also the contributing factor in adsorption performance. The physicochemical properties of raw material are transformed due to the use activating agents. The increasing pattern of total acidic sites (TAC3 > TAC5 > TAC4 > TAC6) is reflected from the increase of H_3_PO_4_ concentration during impregnation. Besides, the concentration of carboxylic groups in TAC3 is the highest among the samples at 0.70 mmol/g. The absence of lactonic groups in TAC2, TAC3, TAC5 and TAC6 indicates that the entire of lactonic groups in twigs have been converted into carboxylic and phenolic groups during chemical activation^19^. TAC1 shows higher basic sites than the other TACs because of the inherent nature of the plant material. Also, there is no chemical agent involved to promote CO_2_ gasification during twigs carbonization at 600 °C. The presence of basic sites in TAC3 and TAC6 is presumably governed in part by anionic phosphate minerals^23^.

Figure S2 shows the pH drift method to determine pH_pzc_ of activated carbons. The interception of coloured lines with the 45° diagonal line represent the pH_pzc_ of activated carbons. The respective values are summarized in Table 4. The decreasing pattern of pH_pzc_ is often associated with the increasing concentration of acidic groups on the adsorbent surface. TAC3 shows a pH_pzc_ of 3.6, while TAC1 exhibits a basic-rich surface with pH_pzc_ of 7.9. The pH_pzc_ of TAC2 and TAC6 are slightly acidic, and close to neutral (pH 7.0), which correlates well with the Boehm titration data. The high value of pH_pzc_ indicates that the surface is negatively charged and may favour the removal of positively charged molecules^19^.

### Adsorption properties

#### Equilibrium adsorption

**Figure 5:**
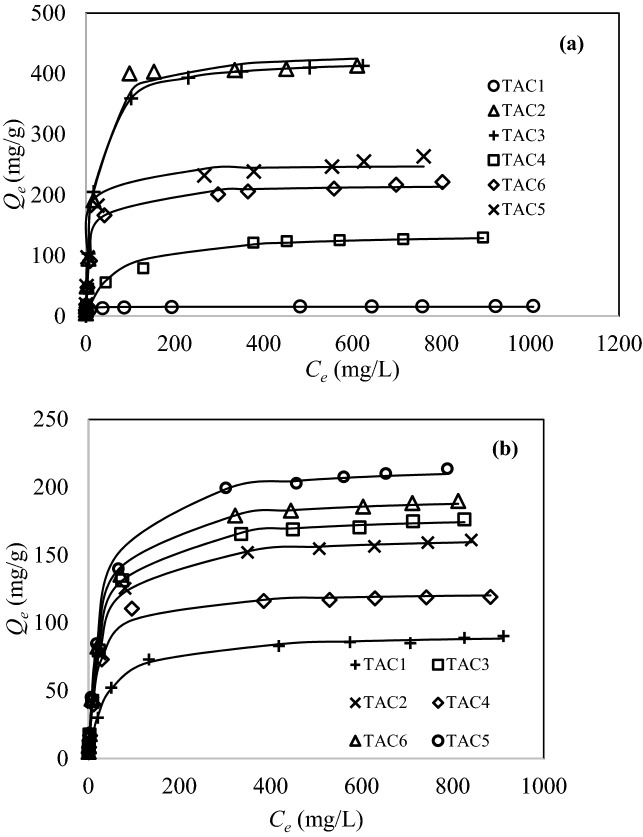
Equilibrium adsorption of (**a**) methylene blue and (**b**) congo red (adsorbent mass: 50 mg, solution volume: 50 mL, dye concentration: 5–1,000 mg/L at room temperature for 72 h; lines were predicted by Langmuir isotherm model).

Figure 5a illustrates the equilibrium adsorption (*Q*_*e*_) of methylene blue onto the activated carbons. For all activated carbons, the equilibrium adsorption increases with increasing the initial concentration (*C*_*e*_) until the saturation point is reached. At equilibrium, the adsorption rate is equal to desorption rate. TAC2 and TAC3 show excellent removal performance at 401 mg/g and 413 mg/g, respectively. The two TACs possess high surface area to encourage interaction probabilities with methylene blue molecules. A slightly bigger methylene blue capacity by TAC3 is due to the high degree of carboxylic groups and total acidic sites on the carbon surface.

The high adsorption capacity signifies the smooth diffusion of methylene blue molecules from bulk solution to the surface of activated carbon. A steep and high adsorption capacity at low concentration indicates a stronger carbon affinity towards dye molecules. Char (TAC1) shows the smallest methylene blue capacity of 16.9 mg/g, which agrees with its inferior surface area as compared with other TACs. The methylene blue capacities of TAC4, TAC5 and TAC6 are 130, 264 and 221 mg/g, respectively. TAC5 exhibits a slightly greater capacity than TAC6 because of higher surface area and smaller pH_pzc_. This signifies the primary role of surface area (pore volume) and pH_pzc_ in methylene blue adsorption by TACs. Generally, the positively charged dye shows a stronger electrostatic attraction with the negatively charged carbon surface, which correlates well with the pH_pzc_ and Boehm titration data.

Figure 5b shows the equilibrium adsorption of congo red onto activated carbons. A small adsorption indicates that the adsorption is concentration-dependent due to limited number of dye molecules in solution at low concentration. The highest congo red capacity is 214 mg/g (TAC5), which is about half that of methylene blue (in mass basis). TAC6 endows a 190 mg/g capacity of congo red. It implies the role of zinc phosphate complex in the performance of TAC5 and TAC6. The formation of complex can be represented as ligand that forms strong covalent bonds with anionic molecules^24^. A better performance of TAC5 than that of TAC6 is due to high H_3_PO_4_ concentration used in activation that results in a higher surface area of 498 m^2^/g.

The negatively charged surface of TAC3 creates electrostatic repulsion that weakens the dispersion forces, hence decreasing the adsorption capacity^25^. Nevertheless, the removal performance of TAC2 (161.1 mg/g) and TAC3 (176.2 mg/g) can still be considered high when compared to that of TAC1 and TAC4. This can be attributed to the high surface area and pore volume of the former. Furthermore, the high adsorption capacity of TAC3 is due to the presence of H_3_PO_4_ that increases the chemisorbed oxygen on the carbon surface^26^ . TAC1 shows a 90.3 mg/g capacity of congo red. TAC4 also possesses a low adsorption capacity (119.1 mg/g) than other treated activated carbons due to poor pore development.

Table 6 show the isotherm constants for methylene blue and congo red adsorption by activated carbons. The isotherm parameters were solved by non-linear regression to provide a mathematically rigorous method from the original equation. The adsorption capacity at molecular level was evaluated in molar basis. TAC3 exhibits a methylene blue capacity of 1.29 mmol/g. From the perspective of molar basis, methylene blue adsorption is greater than congo red adsorption. This could be governed by the smaller molecular size of the former (0.95 nm)^27^ than the latter (2.30 nm)^28^. Thus, methylene blue molecules could easily enter and diffuse through the pore channels of activated carbon. The large molecular size of congo red affect the performance of TACs, whereby the dye molecules can only interact with the mesoporous surface of activated carbon.

**Table 6:** Isotherm constants of methylene blue and congo red adsorption by activated carbons.

Activated carbon	TAC1	TAC2	TAC3	TAC4	TAC5	TAC6
**Methylene blue**						
*Q*_*e,exp*_ (mg/g)	16.9	401	413	130	264	221
*Q*_*e,exp*_ (mmol/g)	0.053	1.25	1.29	0.406	0.825	0.692
**Langmuir model**						
*Q*_*m*_ (mg/g)	15.7	425	438	137	249	217
*Q*_*m*_ (mmol/g)	0.049	1.33	1.37	0.429	0.779	0.678
*b* (L/mg)	0.521	0.053	0.052	0.016	0.152	0.079
SSE	16.5	2,162	2,265	1,790	888	282
R^2^	0.9031	0.9937	0.9937	0.9616	0.9929	0.9978
**Freundlich model**						
*K*_*f*_ ((mg/g)(L/mg)^1/n^)	7.70	77.6	81.5	21.5	58.1	48.7
*n*	8.44	3.62	3.63	3.63	4.25	4.23
SSE	15.35	28,557	32,063	606	7,044	4,789
R^2^	0.9087	0.9210	0.9152	0.9777	0.9450	0.9476
**Redlich–Peterson model**						
*K*	15.5	22.7	22.9	66.1	54.3	19.5
*a*	1.47	0.053	0.052	2.44	0.331	0.11
*g*	0.932	1.00	1.00	0.761	0.932	0.969
SSE	2.59	2,162	2,265	463	146	214
R^2^	0.9845	0.9937	0.9937	0.9825	0.9988	0.9982
**Congo red**						
*Q*_*e,exp*_ (mg/g)	90.3	161	176	119	214	190
*Q*_*e,exp*_ (mmol/g)	0.13	0.231	0.253	0.171	0.307	0.273
**Langmuir model**						
*Q*_*m*_ (mg/g)	92.4	165	180	123	217	194
*Q*_*m*_ (mmol/g)	0.133	0.236	0.258	0.176	0.312	0.278
*b* (L/mg)	0.025	0.037	0.036	0.051	0.035	0.038
SSE	19.9	30.1	38.0	99.0	371	97.5
R^2^	0.9984	0.9994	0.9996	0.9960	0.9972	0.9991
**Freundlich model**						
*K*_*f*_ ((mg/g)(L/mg)^1/n^)	13.1	26.6	28.7	24.6	34.2	31.4
*N*	3.41	3.58	3.53	4.04	3.49	3.56
SSE	931	2,958	3,087	2,662	2,731	3,151
R^2^	0.9313	0.9387	0.9474	0.8967	0.9694	0.9547
**Redlich–Peterson model**						
*K*	2.31	6.02	6.94	6.27	11.0	8.72
*A*	0.025	0.037	0.043	0.051	0.089	0.06
*G*	1.00	1.00	0.98	1.00	0.91	0.95
SSE	19.9	30.1	30.5	99.0	143	39.4
R^2^	0.9984	0.9994	0.9996	0.9960	0.9987	0.9996

From Table 6, the equilibrium data fitted well with Langmuir model with R^2^ in the range of 0.9031 to 0.9978, and 0.9960 to 0.9996 for methylene blue and congo red adsorption, respectively. It can be concluded that the adsorption forms monolayer of molecules onto homogenous carbon surface^19^. It is supported by the smaller sum-of-squared error (SSE) values. The predicted capacities (*Q*_*m*_) of 438 mg/g (TAC3) and 217 mg/g (TAC5) for methylene blue and congo red, respectively are closely tallied with the experimental values.

The Langmuir constant, *b* indicates the affinity of carbon active sites towards dye molecules. From Table 6, TAC1 displays a high *b* value (0.521 L/mg) suggesting a high methylene blue adsorption at low concentration probably due to basic surface that instigates electrostatic interaction. The low *b* but high *Q*_*e*_ reflects the other contributing factors in adsorption. On the contrary, TAC1 shows a small *b* value for congo red removal. The high constant *b* for congo red adsorption is credited to the chemical modification which enhances the textural characteristics of activated carbon. However, the predominance of carboxylic and phenolic groups on carbon surface can create a high degree of H^+^ dissociation towards the repulsion of anionic molecules.

The favourability of dye adsorption was predicted by separation factor, *R*_*L*_ a feature of Langmuir isotherm as shown in Fig. S3. All adsorption data obeyed 0 < *R*_*L*_ < 1, implying that the process is favourable. While, *R*_*L*_ > 1, *R*_*L*_ = 1, and *R*_*L*_ = 0 are attributed to unfavourable, linear, and irreversible adsorption, respectively^29^.

Freundlich model showed the least fitting model for equilibrium adsorption data of methylene blue and congo red by TACs. However, the 1/*n* value lesser than unity agreed well with Langmuir model, while 1/*n* > 1 reflects the adsorption activity between readily adsorbed molecules and free molecules in bulk solution. The Redlich–Peterson model is a hybrid of Langmuir and Freundlich models^30^. At *g* < 1, the large molecules normally restrict the adsorption due to pore clogging, while *g* = 1 simplifies the model into Langmuir equation. A linear Henry’s Law is when *g* = 0. From Table 6, the *K* values are in the range of 15.5–66.1 (methylene blue) and 2.31–11 (congo red). The Redlich–Peterson model supports Freundlich equation when 1/*K* → 0. Conversely, 1/*K* ≠ 0 signifies the dominance of monolayer coverage of dye molecules in adsorption. Table S5 summarizes the comparison of methylene blue and congo red adsorption by wood-based activated carbons. TACs demonstrate comparable and substantial performance, although the surface area (pore volume) may not the sole contributing factor in dyes adsorption.

#### Adsorption kinetics

**Figure 6:**
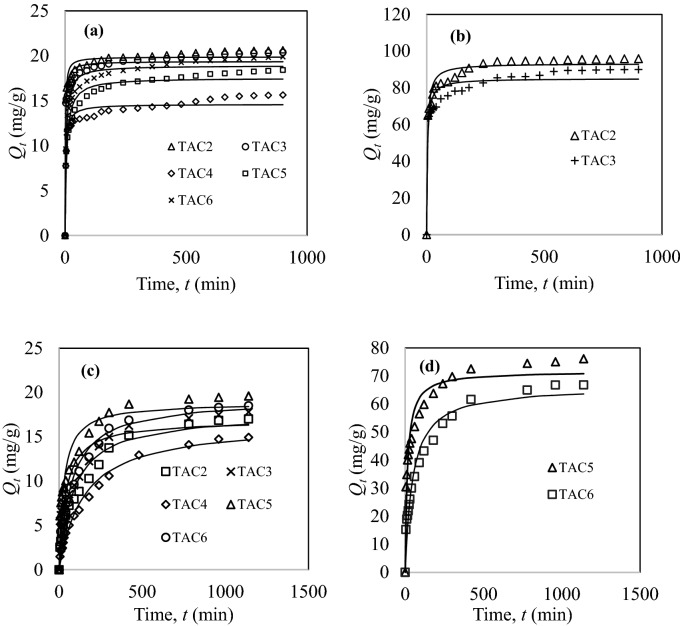
Adsorption kinetics of (**a**,**b**) methylene blue and (**c**,**d**) congo red by TACs at concentrations of (**a**,**c**) 20 mg/L and (**b**,**d**) 100 mg/L (adsorbent mass: 50 mg, solution volume: 50 mL, contact time: 2–540 min at room temperature; lines were predicted by pseudo-second-order model).

Figure 6 illustrates the kinetics of methylene blue and congo red adsorption by TACs. Generally, all curves exhibit a similar convex upward shape of *Q*_*t*_ against *t* but at varying magnitudes at equilibrium. The capacity rapidly increases at the beginning due to plentiful vacant sites on the carbon surface that renders concentration gradient as driving force for adsorption. Thereafter, the adsorption rate subsides because the sites are gradually occupied when approaching equilibrium. Desorption may also take place between the already adsorbed molecules and the free-moving molecules in bulk at prolonged contact time.

From Fig. 6a, TAC2 and TAC3 demonstrate a rapid equilibrium of 3 h, followed by TAC5 and TAC6 (4 h), and TAC4 (5 h) for methylene blue concentration of 20 mg/L. The time taken to attain equilibrium when in contact with 100 mg/L dye solution increases to about 6 h for TAC2 and TAC3, as displayed in Fig. 6b. In Fig. 6c, TAC5 and TAC6 require 5 h to achieve equilibrium for adsorption in 20 mg/L congo red, while a longer contact time was recorded for TAC, TAC3 and TAC4. In Fig. 6d, a 9 h contact time was needed for TAC5 and TAC6 to achieve equilibrium in 100 mg/L congo red. A steep adsorption rate of methylene blue by TAC2 and TAC3 at short contact time is probably due to instantaneous electrostatic interaction of dye molecules with surface acidic functional groups. This effect is more prevalent because methylene blue molecules can easily diffuse through the pores within the carbon matrix.

Table 7 summarizes the kinetics constants of methylene blue and congo red adsorption by TACs. The models demonstrate the rate-limiting step in adsorption. From Table 7, the pseudo-second-order model shows a good correlation with the kinetics data. The close agreement between the calculated *Q*_*e*_ and experimental value shows the applicability of the model to explain the mechanisms of dyes adsorption by TACs. Dyes removal can be described as chemisorption through the exchange of electrons or electrons sharing between the dye molecules and activated carbon^31^. Additionally, the rate-limiting step for dye adsorption is surface adsorption.

**Table 7:** Pseudo-first-order and pseudo-second-order kinetics constants.

Activated carbon	*C*_*o*_ (mg/L)	*Q*_*e,exp*_ (mg/g)	Pseudo-first-order				Pseudo-second-order			
			*Q*_*e*_ (mg/g)	*k*_*1*_ (min^−1^)	SSE	R^2^	*Q*_*e*_ (mg/g)	*k*_*2*_ (g/mg min)	SSE	R^2^
**Methylene blue**										
TAC2	20	19.9	19.4	0.434	33.1	0.9193	19.9	0.039	13.1	0.9680
	100	95.6	90.0	0.147	1,339	0.8639	93.3	0.003	431	0.9555
TAC3	20	19.8	18.8	0.405	53.3	0.8693	19.4	0.032	23.7	0.9418
	100	89.9	81.6	0.189	1607	0.8052	85.1	0.003	718	0.9128
TAC4	20	15.6	14.1	0.209	29.2	0.8903	14.6	0.022	11.7	0.9558
TAC5	20	18.4	16.9	0.088	68.7	0.8695	17.66	0.009	21.1	0.9557
TAC6	20	19.8	18.2	0.150	65.2	0.8676	18.9	0.013	18.5	0.9614
**Congo red**										
TAC2	20	17.0	15.7	0.009	38.5	0.9528	17.7	0.0007	17.2	0.9728
TAC3	20	17.8	15.3	0.019	89.9	0.8460	16.9	0.0015	47.3	0.9070
TAC4	20	14.9	14.2	0.006	15.2	0.9808	16.5	0.0004	6.86	0.9887
TAC5	20	19.6	17.3	0.027	88.0	0.8628	18.9	0.002	39.0	0.9335
	100	76.1	66.9	0.048	1,024	0.8637	71.7	0.001	380	0.9461
TAC6	20	18.5	17.2	0.011	38.1	0.9613	19.3	0.0007	16.5	0.9787
	100	66.8	59.5	0.016	705	0.9245	66.2	0.0003	276	0.9674

The pseudo-first-order equation did not obey the kinetics data for the entire range of contact time but may only applicable for the initial adsorption stage^31^. A high dye concentration induces stronger molecular repulsion and competition for active sites. Consequently, the rate constant, *k*_*2*_ and adsorption rate decreased, resulting in a longer contact time to reach equilibrium. A high *k*_*2*_ value is also associated with the developed porous structure of activated carbon.

Table 8 summarizes the kinetics constants for intraparticle diffusion (Webber-Morris) and Boyd’s models to elucidate the adsorption diffusion mechanism. The adsorption is often controlled by a sequence of four stages: (1) Bulk diffusion—molecules diffuse onto the activated carbon surface from the bulk solution; (2) Film diffusion—molecules diffuse through the boundary layer; (3) Intraparticle diffusion—molecules diffuse from the film surface into the pores of activated carbon; (4) Adsorption—active sites of activated carbon is occupied by molecules^32^. The intercept of intraparticle diffusion plot, *C* signifies that the pore or intraparticle diffusion is not the only rate-limiting step in adsorption. It indicates the boundary layer (film) thickness. A greater *C* implies a thicker film, or film diffusion as dominant rate-limiting step in adsorption^19^.

**Table 8:** Intraparticle diffusion and Boyd’s kinetics constants.

Activated carbon	*C*_*o*_ (mg/L)	*Q*_*e,exp*_ (mg/g)	Intraparticle diffusion model			Boyd’s model		
			*k*_*d*_ (mg/g min)	*C* (mg/g)	R^2^	Intercept	*D*_*i*_ (cm^2^/s)	R^2^
**Methylene blue**								
TAC2	20	19.9	0.147	17.0	0.8118	1.22	5.73 × 10^–9^	0.9355
	100	95.6	0.982	71.9	0.8178	0.95	7.17 × 10^–9^	0.9521
TAC3	20	19.8	0.176	15.9	0.8583	1.02	5.73 × 10^–9^	0.9488
	100	89.9	0.994	64.4	0.9395	0.59	7.17 × 10^–9^	0.9638
TAC4	20	15.6	0.177	10.9	0.7714	0.65	5.73 × 10^–9^	0.9066
TAC5	20	18.4	0.289	11.1	0.8027	0.69	5.02 × 10^–9^	0.9650
TAC6	20	19.8	0.260	13.3	0.7852	0.78	5.73 × 10^–9^	0.9661
**Congo red**								
TAC2	20	17.0	0.489	2.89	0.9340	0.12	4.30 × 10^–9^	0.9829
TAC3	20	17.8	0.424	5.50	0.9313	0.29	4.30 × 10^–9^	0.9855
TAC4	20	14.9	0.461	1.23	0.9636	0.05	4.30 × 10^–9^	0.9670
TAC5	20	19.6	0.438	7.50	0.8788	0.36	5.02 × 10^–9^	0.9830
	100	76.1	1.368	38.5	0.8371	0.55	4.30 × 10^–9^	0.9537
TAC6	20	18.5	0.523	3.78	0.9026	0.17	5.02 × 10^–9^	0.9945
	100	66.8	1.719	18.5	0.9027	0.07	6.45 × 10^–9^	0.8915

Figure S4 shows the intraparticle diffusion curves for methylene blue and congo red removal by TACs at different concentrations. The first region indicates a strong electrostatic interaction between dye molecules and carbon surface; the second region is where the adsorption takes place via molecular diffusion in pores; and the last region signifies the diffusion rate decelerates to a point of equilibrium. In Boyd’s model, the rate-limiting step is attributed to pore diffusion. Effective diffusion coefficient is expressed as, *B* = π*D*_*i*_/*r*^2^, where, *B* is a linear line gradient from *B*_*t*_ versus *t* (s), *D*_*i*_ (cm^2^/s) is the effective diffusion coefficient, and *r* (cm) is the activated carbon radius. According toss^33^, the intraparticle diffusion is the rate-limiting step for *D*_*i*_ in the range of 10^–6^–10^–12^ cm^2^/s. Hence, all TACs studied possess the same rate-limiting step in the adsorption, which is intraparticle diffusion.

#### Adsorption thermodynamics

Figure S5 shows the equilibrium adsorption for (a) methylene blue and (b) congo red onto activated carbons at 30–60 °C. Dye molecules easily diffused from bulk solution into the internal pores at high temperature due to thin film thickness (intraparticle transport pore diffusion)^34^. The adsorption capacity of dyes studied increased with increasing temperature, implying an endothermic nature of adsorption. At high temperature, the solution becomes less viscous and dye molecules gained more kinetics energy to vibrate and surpass the mass transfer resistance, thus accelerating the migration of dye molecules towards the internal pores of carbon matrix^35^. For high dye concentration, the increase in solution temperature aids in weakening the retarding forces against molecular migration. The mobility of dye molecules stimulates more interaction probabilities with carbon sites.

Figure S6 shows the van’t Hoff plots for (a) methylene blue (b) congo red. Table 9 summarizes the thermodynamics parameters of methylene blue and congo red adsorption by activated carbons. The Gibb’s free energy, Δ*G*° becomes more negative at high temperature, suggesting a feasible and spontaneous adsorption process^19^. The positive entropy, Δ*S*° describes the orientation disorder and randomness between dye molecules and carbon surface^36^. From Table 9, the Δ*S*° values are in the range of 112–333 J/mol K (methylene blue) and 66.3–470 J/mol K (congo red). At higher concentration, the dye solution becomes more dispersed, rendering the molecules more freedom of arrangement, hence increasing the entropy.

**Table 9:** Thermodynamics parameters of methylene blue and congo red adsorption by activated carbons.

Activated carbon	*C*_*o*_ (mg/L)	T (K)	*K*_*d*_	ln *K*_*d*_	Δ*G*° (kJ/mol)	Δ*H*° (kJ/mol)	Δ*S*° (J/mol K)
**Methylene blue**							
TAC2	20	303	129.9	4.87	– 12.3	88.2	333.4
		313	699.0	6.55	– 17.0		
		323	1542	7.34	– 19.7		
		333	3,257	8.09	– 22.4		
	100	303	9.02	2.20	– 5.54	68.7	245.2
		313	20.2	3.01	– 7.82		
		323	62.9	4.14	– 11.1		
		333	94.1	4.54	– 12.6		
TAC3	20	303	13.7	2.62	– 6.60	27.3	112.3
		313	18.7	2.93	– 7.62		
		323	38.6	3.65	– 9.81		
		333	31.6	3.45	– 9.56		
	100	303	10.2	2.33	– 5.86	39.6	150.3
		313	21.0	3.05	– 7.93		
		323	23.2	3.14	– 8.44		
		333	47.9	3.87	– 10.7		
**Congo red**							
TAC5	20	303	5.55	1.71	– 4.32	51.2	182.8
		313	10.3	2.34	– 6.08		
		323	16.4	2.80	– 7.51		
		333	36.5	3.60	– 9.96		
	100	303	2.46	0.90	– 2.27	17.7	66.3
		313	3.50	1.25	– 3.26		
		323	3.59	1.28	– 3.43		
		333	4.94	1.60	– 4.42		
TAC6	20	303	6.94	1.94	– 4.88	138	470
		313	20.2	3.01	– 7.82		
		323	231	5.44	– 14.6		
		333	749	6.62	– 18.3		
	100	303	1.64	0.50	– 1.25	18.9	66.9
		313	2.38	0.87	– 2.25		
		323	2.77	1.02	– 2.74		
		333	3.29	1.19	– 3.30		

### Adsorption mechanisms

The possible mechanisms of methylene blue adsorption by TAC3 consist of ion-exchange, hydrogen bonding, π-π conjugation, electrostatic attraction, and physical adsorption. The π–π conjugation is a lateral attraction between aromatic ring of dye molecule and graphitic (aromatic) layer of carbon^37^, while physisorption is generally tied-up with van der Waals forces because of the well-developed porous structure of activated carbon. From the equilibrium and kinetics data, TAC3 exhibits chemisorption by electron withdrawn containing π-electron acceptors such as ketone, aldehyde, and benzene groups. In addition, N–H group of methylene blue can establish hydrogen bonding with carboxylic (-COOH) and hydroxyl groups (-OH) in TAC3 surface. Also, the carboxylic groups dissociate the protons and become negatively charged to offer electrostatic attraction with methylene blue molecules. Accordingly, the solution pH slightly decreased due to the liberation of H^+^ ions^38^. Electrostatic interaction also exists between acidic carbon sites and basic methylene blue dye when the solution is higher than pH_pzc_, at which the surface is surrounded by excess OH^−^. Besides, the Si–OH group present in TAC3 possibly prompts the n-π interaction with methylene blue. The presence of Si–OH group in TAC3 (Fig. 4) enhances the removal of methylene blue in basic environment^39^.

Complex interaction of Zn_3_(PO_4_)_2_·4H_2_O with congo red is the main mechanism for TAC5. The hydrated salt that holds a positively charged Zn^2+^ allows electrostatic interaction with anionic dye. According to^40^, ion–dipole interaction may take place from the interaction of Zn^2+^ with polar molecules (partial negatively charged oxygen in congo red). Additionally, amine group in congo red can also form hydrogen bonding with phosphate ion (PO_4_^3−^). These two interactions are attributed to hydrophilic adsorption. Similar mechanism has been proposed for congo red removal by calcium hydroxyapatite^41^.

## Conclusion

The *Pterocarpus indicus* twigs-derived activated carbons were successfully prepared by a series of impregnation ratios using composite of zinc chloride and/or phosphoric acid. The activated carbons display unique physicochemical characteristics for selective dyes removal in water. TAC3 exhibits the preference towards cationic methylene blue, while TAC5 demonstrates excellent performance for anionic congo red removal. The maximum adsorption capacities were recorded as 438 mg/g (TAC3) and 217 mg/g (TAC5), respectively. Multiple mechanisms may involve in dyes adsorption. The adsorption of dyes by TACs are favourable and spontaneous at high temperature. To conclude, *Pterocarpus indicus* twigs is a promising candidate of activated carbon for wastewater treatment, and the activation strategy via H_3_PO_4_/ZnCl_2_ composite chemical activation has been established to yield selective and outstanding removal of dyes in water.

## Supplementary information

Supplementary Information.
