# Supplementary material for: Twigs-derived activated carbons via H3PO4/ZnCl2 composite activation for methylene blue and congo red dyes removal
Source: Sci Rep. 2020 Aug 20;10:14050. doi: 10.1038/s41598-020-71034-6 (PMC7441319; doi:10.1038/s41598-020-71034-6)
Supplement: Supplementary file 1 — Supplementary Information. [file 41598_2020_71034_MOESM1_ESM.docx]

**Supplementary Data**

**Twigs-derived activated carbons via H_3_PO_4_/ZnCl_2_ composite activation for methylene blue and congo red dyes removal**

Muhammad Hadzirun Muhamad Zubir^a,b^, Muhammad Abbas Ahmad Zaini^a,b.^*

^a^ Centre of Lipids Engineering & Applied Research (CLEAR), Ibnu-Sina Institute for Scientific & Industrial Research, Universiti Teknologi Malaysia, 81310 UTM Johor Bharu, Johor, Malaysia

^b^ School of Chemical & Energy Engineering, Faculty of Engineering, Universiti Teknologi Malaysia, 81310 UTM Johor Bahru, Johor, Malaysia

*Corresponding author: abbas@cheme.utm.my

**Table S1** Adsorption models and thermodynamics parameters.

| Model | Equation | Description | Reference |
| --- | --- | --- | --- |
| *Isotherm* | |  |  |
| 1. Langmuir  $Q_{e}=\frac{Q_{m}bC_{e}}{1+ bC_{e}}$ | (S1) | *Q_e_* (mg/g): Equilibrium adsorption capacity  *Q_m_* (mg/g): Maximum adsorption capacity (monolayer adsorption)  *b* (L/mg): Affinity of adsorption  *C_e_* (mg/L): Equilibrium concentration in solution | [1] |
|  |  |  |  |
| 2. Freundlich  $Q_{e}= K_{F}{C_{e}}^{\frac{1}{n}}$ | (S2) | *K_F_* (mg/g)(L/mg)^1/^*^n^*: Freundlich adsorption constant  *n*: Adsorption site intensity (dimensionless) | [2] |
|  |  |  |  |
| 3. Redlich-Peterson  $Q_{e}=\frac{K_{R}C_{e}}{{1+ a_{R}C_{e}}^{g}}$ | (S3) | *K_R_* (L/g): Isotherm constant  *a_R_* (L/mg)*^g^*: Isotherm constant  *g*: Isotherm exponent | [3] |
|  | |  |  |
| *Kinetics* | |  |  |
| 1. Pseudo-first-order  $Q_{t}=Q_{e}[1-\exp\left( -k_{1}t \right)]$ | (S4) | *Q_t_*  (mg/g): Adsorption capacity at time *t*  *Q_e_* (mg/g): Equilibrium adsorption capacity  *k_1_* (min^-1^): Pseudo-first-order rate constant  *t* (min): contact time | [4] |
|  |  |  |  |
| 2. Pseudo-second-order  $Q_{t}=\frac{k_{2}{Q_{e}}^{2}t}{1+k_{2}Q_{e}t}$ | (S5) | *k_2_* (g/mg.min): Pseudo-second-order rate constant  *t* (min): contact time | [5] |
|  |  |  |  |
| 3. Intraparticle diffusion  $Q_{t}= k_{d}t^{0.5}+C$ | (S6) | *k_d_* (mg/g.min^0.5^): Intraparticle diffusion rate constant  *C* (mg/g): Intercept of intraparticle diffusion plot | [6] |
|  |  |  |  |
| 4. Boyd’s  $B_{t}=-0.4977-\ln\begin{aligned} \left( 1-F \right) \end{aligned}$  for *F* > 0.85  $B_{t}=\left( \sqrt{\pi}-\sqrt{\pi-\left( \frac{\pi^{2}F}{3} \right)} \right)^{2}$  for *F* < 0.85  *B* = π*D_i_*/*r*^2^ | (S7)  (S8)  (S9) | *F = Q_t_/Q_e_*: Fraction of solute adsorbed at time, *t* (min)  *B* (s^-1^): Slope of *B_t_* versus *t* (s) graph  *D_i_* (cm^2^/s): Effective diffusion coefficient  *r* (cm): Radius particle | [7] |
|  |  |  |  |
| *Thermodynamics* | |  |  |
| 1. Gibb’s free energy  $\Delta G^{\circ}=\Delta H^{\circ}-T$ ΔS°  $\ln K_{d}=\frac{\Delta S^{\circ}}{R}-\frac{\Delta H^{\circ}}{\mathrm{RT}}$  $\Delta G^{\circ}=-RT\ln K_{d}$ | (S10)  (S11)  (S12) | Δ*G*° (kJ/mol): Free energy change  Δ*H*° (kJ/mol): Enthalpy change  Δ*S*° (J/mol.K): Entropy change  *K_d_*: Adsorption distribution coefficient  *T* (K): Absolute temperature in Kelvin  *R* (J/mol.K): Universal gas constant (8.314 J/mol.K) | [8] |

**Table S2** Proximate analysis.

| Sample | Moisture content (%) | Ash content (%) | Volatile matter (%) | Fixed carbon (%) |
| --- | --- | --- | --- | --- |
| TRM | 8.00 | 10.0 | 72.0 | 10.0 |
| imTRM2 | 10.0 | 23.0 | 49.0 | 14.0 |
| imTRM3 | 5.00 | 40.0 | 29.0 | 26.0 |
| imTRM4 | 9.00 | 45.0 | 20.0 | 13.0 |
| TAC2 | 23.0 | 38.0 | 4.00 | 33.0 |
| TAC3 | 28.0 | 35.0 | 2.00 | 34.0 |
| TAC4 | 5.00 | 76.0 | 6.00 | 12.0 |

**Table S3** Elemental composition of some activated carbon precursors.

| Raw material |  |  | Wt. (%) |  |  | Reference |
| --- | --- | --- | --- | --- | --- | --- |
|  | C | H | N | S | O |  |
| *Pterocarpus indicus* (TRM) | 46.3 | 6.87 | 0.265 | 0.041 | 46.5 | Present study |
| Paulownia wood | 45.8 | 6.29 | 0.40 | - | 47.5 | [9] |
| Pine wood | 45.0 | 6.40 | 1.30 | - | 47.3 | [10] |
| Barley straw | 45.4 | 6.10 | 0.70 | 0.07 | 41.9 | [11] |
| Palm kernel shell | 48.9 | 5.21 | 0.07 | 0.19 | 45.6 | [12] |
| Bamboo hydrochar | 49.3 | 6.23 | 0.19 | - | 44.3 | [13] |

**Table S4** Porosity characteristics of TAC3 and the comparison with similar activated carbons.

| Raw material | Activation time (min) | Activating agent | BET surface area  (m^2^/g) | Total pore volume  (cm^3^/g) | Meso-porosity (%) | Reference |
| --- | --- | --- | --- | --- | --- | --- |
| *Pterocarpus indicus* (TAC3) | 90 | H_3_PO_4_ | 1445 | 0.730 | 67.5 | This study |
| Pine sawdust | 120 | CO_2_ | 352 | 0.194 | 8.25 | [14] |
| *Havea braziliansis* | 120 | K_2_CO_3_ | 686 | 0.290 | 62.1 | [15] |
| Coconut frond | 120 | H_3_PO_4_ | 484 | 0.36 | 80.6 | [16] |
| *Tamarix hispida* wood | 120 | NH_4_Br | 1006 | 0.621 | - | [17] |

**Table S5** Adsorption capacity of methylene blue and congo red by various activated carbons.

| Material | Activating agent | BET surface area (m^2^/g) | Mesoporosity (%) | Langmuir affinity, *b* (L/mg) | Monolayer adsorption  capacity, *Q_m_* (mg/g) | Reference |
| --- | --- | --- | --- | --- | --- | --- |
| Methylene blue | |  |  |  |  |  |
| *Pterocarpus indicus* (TAC3) | H_3_PO_4_ | 1445 | 67.5 | 0.052 | 438 | This work |
| *Ananas comosus* (Pineapple) | ZnCl_2_ | 915 | 72.5 | 0.075 | 288 | [18] |
| Sewage sludge | CO_2_ | 260 | - | 0.177 | 59.5 | [89] |
| *Mauritia flexuosa* L. (Buriti shells) | ZnCl_2_ | 843 | 25.0 | 3.11 | 275 | [20] |
| Congo red | |  |  |  |  |  |
| *Pterocarpus indicus* (TAC5) | ZnCl_2_ + H_3_PO_4_ | 498 | 66.2 | 0.035 | 217 | This work |
| Date pits | HNO_3_ | 1160 | - | 0.013 | 105 | [21] |
| Coffee waste | CH_3_CO_2_K | 220 | 56.0 | 0.15 | 90.9 | [22] |
| Sycamore bark | ZnCl_2_ | 1393 | 91.6 | 0.499 | 56.4 | [23] |

**(a)**

**(b)**

**Figure S1** (a) N_2_ adsorption-desorption and (b) pore size distribution of TACs.

**Figure S2** pH_pzc_ of activated carbons (adsorbent mass: 100 mg, solution volume: 50 mL, pH: 2.5 - 10.5 mg/L at room temperature for 24 h).

**(a)**

**Figure S3** Separation factor for (a) methylene blue and (b) congo red adsorption by TACs.

**(a)**

**Figure S4** Intraparticle diffusion model for (a and b) methylene blue and (c and d) congo red adsorption onto activated carbons at concentrations of (a and c) 20 mg/L and (b and d) 100 mg/L.

**(a)**

**Figure S5** Effect of temperature of equilibrium adsorption of (a) methylene blue and (b) congo red by activated carbons (adsorbent mass: 50 mg, solution volume: 50 mL, solution temperature: 30 - 60 °C for 72 h).

**Figure S6** The van’t Hoff thermodynamics relation for (a) methylene blue and (b) congo red adsorption.

**References**

[1] Langmuir, I. The constitution and fundamental properties of solids and liquids. *Journal of American Chemical Society*. **38**, 2221–2295 (1916).

[2] Freundlich, H.M.F. Over the adsorption in solution. *Journal of Physical Chemistry*. **57**, 385–471 (1906).

[3] Redlich, O. & Peterson, D.L. A Useful Adsorption Isotherm. *The Journal of Physical Chemistry*. **63**, 1024–1024 (1959).

[4] Lagergren, S. About the theory of so-called adsorption of soluble substances. *Handlingar*, **24**, 1-39 (1898).

[5] Ho, Y.S. & McKay, G. A comparison of chemisorption kinetic models applied to pollutant removal on various sorbents. *Process Safety and Environmental Protection*. **76**, 332–340 (1998).

[6] Weber, W.J. & Morris, J.C. Kinetics of adsorption on carbon from solutions. *Journal of the Sanitary Engineering Division*. **89**, 31–60 (1963).

[7] Boyd, G.E., Adamson, A.W. & Myers, L.S. The exchange adsorption of ions from aqueous solutions by organic zeolites, II, Kinetics. *Journal of the American Chemical Society*. **69**, 2836–2848 (1947).

[8] Lima, E.C., Hosseini-Bandegharaei, A., Moreno-Piraján, J.C. & Anastopoulos, I. A critical review of the estimation of the thermodynamic parameters on adsorption equilibria. Wrong use of equilibrium constant in the Van't Hoof equation for calculation of thermodynamic parameters of adsorption. *Journal of Molecular Liquids*. **273**, 425–434 (2019).

[9] Yorgun, S. & Yıldız, D. Preparation and characterization of activated carbons from Paulownia wood by chemical activation with H_3_PO_4_. *Journal of the Taiwan Institute of Chemical Engineers*. 53, 122–131 (2015).

[10] Ronewicz, K., Kluska, J., Heda, Ł. & Kardaś, D. Chemical and physical properties of pine wood during pyrolysis. *Drvna industrija*. 68, 29-36 (2017).

[11] Pallarés, J., González-Cencerrado, A. & Arauzo, I. Production and characterization of activated carbon from barley straw by physical activation with carbon dioxide and steam. *Biomass and Bioenergy*. 115, 64–73 (2018).

[12] Rugayah, A.F., Astimar, A.A. & Norzita, N. Preparation and characterisation of activated carbon from palm kernel shell by physical activation with steam. *Journal of Oil Palm Research*. 26(3), 251-264 (2014).

[13] Qian, W., Luo, X., Wang, X., Guo, M. & Li, B. Removal of methylene blue from aqueous solution by modified bamboo hydrochar. *Ecotoxicology and Environmental Safety*. 157, 300–306 (2018).

[14] Nowicki, P. & Pietrzak, R. Carbonaceous adsorbents prepared by physical activation of pine sawdust and their application for removal of NO_2_ in dry and wet conditions. *Bioresource Technology*. 101, 5802–5807 (2010).

[15] Krishnan, K.A., Sreejalekshmi, K.G. & Varghese, S. Adsorptive retention of citric acid onto activated carbon prepared from Havea braziliansis sawdust: kinetic and isotherm overview. *Desalination*. 257, 46–52 (2010).

[16] Njoku, V.O., Islam, M.A., Asif, M. & Hameed, B.H. Preparation of mesoporous activated carbon from coconut frond forthe adsorption of carbofuran insecticide. *Journal of Analytical and Applied Pyrolysis*. 110, 172–180 (2014).

[17] Khademi, Z., Ramavandi, B. & Ghaneian, M.T. The behaviors and characteristics of a mesoporous activated carbon prepared from *Tamarix hispida* for Zn(II) adsorption from wastewater. *Journal of Environmental Chemical Engineering*. 3, 2057–2067 (2015).

[18] Mahamad, M.N., Zaini, M.A.A. & Zakaria, Z.A. Preparation and characterization of activated carbon from pineapple waste biomass for dye removal. *International Biodeterioration & Biodegradation*. 102, 274-280 (2015).

[19] Sierra, I., Iriarte-Velasco, U., Cepeda, E.A., Gamero, M. & Aguayo, A.T. Preparation of carbon-based adsorbents from the pyrolysis of sewage sludge with CO_2_. Investigation of the acid washing procedure. *Desalination and Water Treatment*. 57(34), 16053–16065 (2016).

[20] Pezoti, O. *et al*. Adsorption studies of methylene blue onto ZnCl_2_-activated carbon produced from buriti shells (*Mauritia flexuosa* L.). *Journal of Industrial and Engineering Chemistry*. 20, 4401–4407 (2014).

[21] Belhachemia, M. & Addoun, F. Adsorption of congo red onto activated carbons having different surface properties: studies of kinetics and adsorption equilibrium. *Desalination and Water Treatment*. 37, 122–129 (2011).

[22] Lafi. R., Gzara, L., Lajimi, R.H. & Hafiane, A. Treatment of textile wastewater by a hybrid ultrafiltration/electrodialysis process. *Chemical Engineering and Processing - Process Intensification*. 132, 105-113 (2018).

[23] Cong, L., Feng, L., Wei, X., Jin, J. & Wu, K. Study on the Adsorption Characteristics of Congo Red by Sycamore Bark Activated Carbon. *Environment Technology Resources*. 1, 64–69 (2017).
